# Optical Characterization of As_x_Te_100−x_ Films Grown by Plasma Deposition Based on the Advanced Optimizing Envelope Method

**DOI:** 10.3390/ma13132981

**Published:** 2020-07-03

**Authors:** Dorian Minkov, George Angelov, Radi Nestorov, Aleksey Nezhdanov, Dmitry Usanov, Mikhail Kudryashov, Aleksandr Mashin

**Affiliations:** 1 College of Energy and Electronics, Technical University of Sofia, 2140 Botevgrad, Bulgaria; 2 Department of Microelectronics, Technical University of Sofia, 1000 Sofia, Bulgaria; angelov@ecad.tu-sofia.bg (G.A.); rnn@ecad-tu-sofia.bg (R.N.); 3 Laboratory of Functional Nanomaterials, Lobachevsky University, 603950 Nizhny Novgorod, Russia; usanov@phys.unn.ru (D.U.); kudryashov@phys.unn.ru (M.K.); mashin@phys.unn.ru (A.M.)

**Keywords:** advanced optimizing envelope method, optical characterization, superior accuracy, As_x_Te_100−x_ films, light scattering, partial coherence, surface roughness

## Abstract

Three As_x_Te_100__−__x_ films with different x and dissimilar average thickness $\bar{d}$ are characterized mainly from one interference transmittance spectrum *T*(*λ* = 300 to 3000 nm) of such film on a substrate based on the advanced optimizing envelope method (AOEM). A simple dual transformation of *T*(*λ*) is proposed and used for increasing the accuracy of computation of its envelopes *T*_+_(*λ*) and *T*_−_(*λ*) accounting for the significant glass substrate absorption especially for *λ* > 2500 nm. The refractive index *n*(*λ*) of As_40_Te_60_ and As_98_Te_2_ films is determined with a relative error <0.30%. As far as we know, the As_80_Te_20_ film is the only one with anomalous dispersion and the thickest, with estimated $\bar{d}$ = 1.1446 nm, ever characterized by an envelope method. It is also shown and explained why the extinction coefficient *k*(*λ*) of any of the three As_x_Te_100__−__x_ films is computed more accurately from the quantity *T*_i_(*λ*) = [*T*_+_(*λ*)*T*_−_(*λ*)]^0.5^ compared to its commonly employed computation from *T*_+_(*λ*). The obtained results strengthen our conviction that the AOEM has a capacity for providing most accurate optical characterization of almost every dielectric or semiconductor film with $\bar{d}$ > 300 nm on a substrate, compared to all the other methods for characterization of such films only from *T*(*λ*).

## 1. Introduction

Thin films have widespread applications in optics, optoelectronics and magneto-optics [1,2], as the films can be deposited by different techniques, using various technology regimes [3,4,5]. The ever increasing requirements regarding the performance of devices containing such films determine the necessity for increasing the accuracy of optical characterization of the films.

Most optical methods for characterization of thin films use at least one normal incidence transmittance spectrum *T*(*λ*) of a specimen consisting of the studied film deposited on a light transmitting substrate, such as glass [6,7]. Such methods can be classified into three groups: dispersion methods, hybrid methods, and interference methods [8]. The dispersion methods, including all spectroscopic ellipsometry methods, implement a dispersion model represented by a formula containing at least one of the components of the complex refractive index [9,10]. However, dispersion models are usually not accurate for doped films [11,12], organic films [13,14], and mechanically stressed films [15]. In the hybrid methods, a dispersion model or independent measurement of the film thickness is used, which is often insufficiently accurate, together with *T*(*λ*) [13,16].

The interference methods employ at least one interference transmittance or reflectance spectrum, i.e., a spectrum containing interference pattern with several maxima and minima due to light interference in the film [17,18]. The most popular interference method is the founding envelope method (FEM) of Swanepoel [19], which employs only one normal incidence transmittance spectrum *T*(*λ*) of the specimen, and is the most cited method for optical characterization of a thin film, with over 4400 citations [20]. The FEM also uses a computed smoothed spectrum *T*_sm_(*λ*) of *T*(*λ*), as well as its upper envelope *T*_+_(*λ*) and lower envelope *T*_−_(*λ*). Notwithstanding its popularity, the FEM from [19] and its modifications [21,22,23,24,25,26] assume that the film has uniform thickness $\bar{d}$ and the substrate is transparent, which can result in significant errors in film characterization. Both the thickness non-uniformity ∆d ≥ 0 of the film over the light spot and the substrate absorption have been taken into account in the improved envelope method (IEM) [27]. However, it uses three subjectively chosen parameters which can also lead to significant film characterization errors.

**Figure 1:**
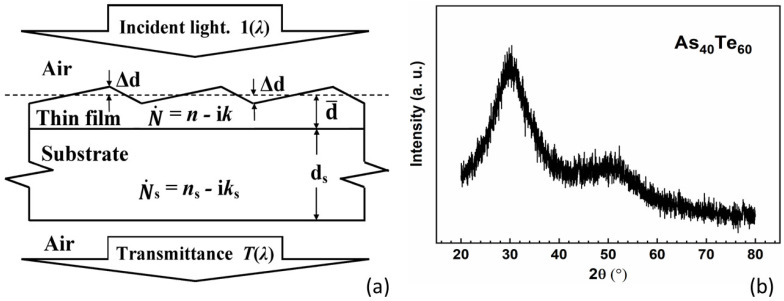
(**a**) A sketch of a homogeneous thin film on a substrate specimen, and its main optical characteristics [29]. (**b**) X-ray diffraction (XRD) image for a plasma deposited As_40_Te_60_ film on a glass substrate.

The optimizing envelope method (OEM) [28] determines optimized values of the above mentioned three parameters, and employs them in the computation of $\bar{d}$, ∆d, and the spectral dependencies of the refractive index *n*(*λ*) and the extinction coefficient *k*(*λ*) of the film illustrated in Figure 1.

The smoothed normal incidence transmittance spectrum *T*_sm_ (*λ*) of the specimen from Figure 1 was formulated in [28] as:

(1) $$T_{\mathrm{sm}}\left(\lambda \right)=\frac{1}{\varphi_{2}-\varphi_{1}}\int_{\varphi_{1}}^{\varphi_{2}}T_{u}(\varphi )d\varphi =\frac{{\left(\tau_{a,f}\tau_{f,s}\tau_{s,a}\right)}^{2}x_{s}}{\varphi_{2}-\varphi_{1}}\int_{\varphi_{1}}^{\varphi_{2}}{\frac{x_{}d\varphi }{a_{1}-b_{1}\cos\left(\varphi \right)+c_{1}\sin\left(\varphi \right)}}_{},$$

where:

$$\varphi =4\pi nd/\lambda ,\varphi_{1}=4\pi n(\bar{d}-\Delta d)/\lambda ,\varphi_{2}=4\pi n(\bar{d}+\Delta d)/\lambda ,x=\exp(-4\pi kd/\lambda ),\;x_{s}=\exp(-4\pi k_{s}d_{s}/\lambda ),a_{1}=1-{\left(\rho_{a,f}\rho_{s,a}xx_{s}\right)}^{2}+\rho_{f,s}{}^{2}(\rho_{a,f}{}^{2}x^{2}-\rho_{s,a}{}^{2}x_{s}{}^{2}),$$

$$b_{1}=2\rho_{a,f}\rho_{f,s}\rho_{s,a}x_{}[\rho_{s,a}x_{s}{}^{2}\cos\Delta_{2}-\rho_{s,a}{}^{-1}\cos\Delta_{1}]{,}_{}c_{1}=2\rho_{a,f}\rho_{f,s}\rho_{s,a}x_{}[\rho_{s,a}x_{s}{}^{2}\sin\Delta_{2}-\rho_{s,a}{}^{-1}\sin\Delta_{1}],$$

$$\tau_{a,f}\tau_{f,s}\tau_{s,a}=\frac{8}{\sqrt{{\left(n+1\right)}^{2}+k^{2}}}\sqrt{\frac{n^{2}+k^{2}}{{\left(n+n_{s}\right)}^{2}+{\left(k+k_{s}\right)}^{2}}}\sqrt{\frac{n_{s}{}^{2}+k_{s}{}^{2}}{{\left(n_{s}+1\right)}^{2}+k_{s}{}^{2}},}$$

$$\rho_{a,f}=\sqrt{\frac{{\left(n-1\right)}^{2}+k^{2}}{{\left(n+1\right)}^{2}+k^{2}}},\rho_{f,s}=\sqrt{\frac{{\left(n-n_{s}\right)}^{2}+{\left(k-k_{s}\right)}^{2}}{{\left(n+n_{s}\right)}^{2}+{\left(k+k_{s}\right)}^{2}}},\rho_{s,a}=\sqrt{\frac{{\left(n_{s}-1\right)}^{2}+k_{s}{}^{2}}{{\left(n_{s}+1\right)}^{2}+k_{s}{}^{2}}},$$

$$\begin{array}{l} \Delta_{1}=\tan{}^{-1}\left(\frac{2k}{n^{2}+k^{2}-1}\right)+\pi +\tan{}^{-1}\left[\frac{2(kn_{s}-k_{s}n)}{n^{2}-n_{s}{}^{2}+k^{2}-k_{s}{}^{2}}\right],\\ \Delta_{2}=\tan{}^{-1}\left(\frac{2k}{n^{2}+k^{2}-1}\right)+\pi -\tan{}^{-1}\left[\frac{2(kn_{s}-k_{s}n)}{n^{2}-n_{s}{}^{2}+k^{2}-k_{s}{}^{2}}\right]; \end{array}$$

where *T*_u_(*λ*) represents the transmittance of a uniform film on the same substrate, *x*(*λ*) is the absorbance of the film, and the subscript ‘s’ refers to the respective known substrate characteristics. It is assumed in Equation (1) that the film thickness *d* has a continuous uniform distribution in the interval ($\bar{d}$ − ∆d, $\bar{d}$ + ∆d) over the light spot on the film surface, as the light passing through the film is considered to be coherent.

In an advanced version of the OEM (AOEM) [29] was pointed out that partial coherence of light, due to both light scattering from the film and finite slit width, results in slight shrinkage of the interference pattern. To offset these effects, it was proposed in [29] that the smoothed spectrum *T*_sm_(*λ*) should be computed by “external smoothing” of *T*(*λ*), to correspond to the coherence of light passing through the film assumed in Equation (1), instead of the commonly used “internal smoothing” of *T*(*λ*).

The envelopes *T*_+_(*λ*) and *T*_−_(*λ*) of *T*_sm_(*λ*) were computed in [29] by combining the advantages of including extra points for the interpolation of both envelopes [30], with these of using iterations [31], however, implying substrate transparency for all *λ*. Moreover, points *T*_+_(*λ*_t_) and *T*_−_(*λ*_t_) were adjusted in the spectral region with *x*_s_(*λ*) < 1 to take into account the substrate non-transparency there [29]. The following accurate formula for the envelope *T*_+_(*λ*) was also presented in [29]:

(2) $$T_{+}(\lambda )=\frac{1}{\varphi_{2}{}_{{}_{+}}-\varphi_{1_{+}}}\int_{\varphi_{1_{+}}}^{\varphi_{2_{+}}}T_{u}(\varphi_{+})d\varphi_{+}=\frac{{\left(\tau_{a,f}\tau_{f,s}\tau_{s,a}\right)}^{2}x_{s}}{\varphi_{2}{}_{{}_{+}}-\varphi_{1_{+}}}\int_{\varphi_{1_{+}}}^{\varphi_{2_{+}}}{\frac{x_{}d\varphi_{+}}{a_{1}-b_{1}\cos\left(\varphi_{+}\right)+c_{1}\sin\left(\varphi_{+}\right)}}_{},$$

where $\varphi_{+}=4\pi n(d-\bar{d})/\lambda ,\varphi_{1_{+}}=-4\pi n\Delta d/\lambda ,\varphi_{2_{+}}{=}_{}4\pi n\Delta d/\lambda_{}.$ Equations (1) and (2) have been derived assuming *n*^2^(*λ*) > *n*_s_^2^(*λ*) >> *k*^2^(*λ*) and *n*_s_^2^(*λ*) >> *k*_s_^2^(*λ*) [27], as these relationships are usually satisfied in the UV/VIS/NIR spectral region for a thin dielectric or semiconductor film with $\bar{d}$ = (300,5000) nm on a glass substrate [24].

Main advantage of the envelope methods (EM) is that they do not employ and dispersion model, as a result of using the interference fringes equation [19,21,22,23,24,25,26,27,28,29]:

(3) $$2_{}n{\left(\lambda_{t}\right)}_{}\bar{d}=m_{i}(\lambda_{t})\lambda_{t}{\left(i\right)}_{}{}_{}\{\begin{array}{c} m_{i}\geq 1-\mathrm{integer}\;\mathrm{for}\;\mathrm{all}\;\mathrm{tangency}\;\mathrm{wavelengths}\;\lambda_{t_{+}}{}_{}\;\mathrm{from}\;\mathrm{the}\;\mathrm{envelope}\;T_{+}(\lambda )\\ m_{i}{}_{}\geq 1/2_{}-\mathrm{half}-\mathrm{integer}\;\mathrm{for}\;\mathrm{all}\;\mathrm{tangency}\;\mathrm{wavelengths}\;\lambda_{t_{-}}\;\mathrm{from}\;\mathrm{the}\;\mathrm{envelope}\;T_{-}(\lambda ). \end{array}$$

The tangency wavelengths *λ*_t_(i) in Equation (3) represent the tangency points *T*_sm_(*λ*_t_) between *T*_sm_(*λ*) and its two envelopes, where ‘i’ is a positive integer showing the number of the ‘i-th’ extremum of *T*_sm_(*λ*) counted from 1 closest from the higher wavelengths end, and *m*_i_(*λ*_t_) is the interference order.

The AOEM is executed in two stages, as at the end of its first stage are computed both the average thickness $\bar{d}$, and the thickness non-uniformity ∆d ≥ 0 of the film over the light spot, as well as the first interference order *m*_1_(max(*λ*_t_)) [28,29]. This is achieved by minimization of an error metric. The performance of seven possible error metrics was compared in [32], as it was shown there that using the AOEM and the error metric:

(4) $$\frac{1}{N}\sqrt{\frac{\sum_{i=1}^{N_{}}{\left\{{\bar{d}}_{}{-}_{}{\bar{d}}_{0}\left[\lambda_{t}(i)\right]\right\}}^{2}}{N_{}}}=\frac{\delta d}{N}\geq 0_{}{{}_{}}_{}{}_{}$$

which provides $\bar{d}$ with an accuracy of 0.06% independent from the characteristics of model homogeneous films. In Equation (4), $\bar{d}$_0_(*λ*_t_(i)) are estimated values of $\bar{d}$, *N* is an optimized number of adjacent tangency wavelengths *λ*_t_(i) participating in the computations, and δd is the root mean square deviation of $\bar{d}$_0_(*λ*_t_(i)) from $\bar{d}$. The employment of larger optimized *N* in Equation (4) and more accurate respective values of $\bar{d}$ and *m*_1_ make possible accurate characterization of thicker films by the AOEM in comparison with the FEM and the IEM [5,29].

In the second stage of the AOEM the refractive index *n*(*λ*) of the film is computed by optimized curve fitting over approximated values *n*_0_{*Λ*_t_} of *n*{*Λ*_t_} calculated by using Equation (3), where the wavelengths set {*Λ*_t_} contains all wavelengths used in the interpolation of the envelopes of *T*_sm_(*λ*), including all *λ*_t_ [29]. The extinction coefficient *k*(*λ*) of the film can be determined as:

(5) $$k\left[T_{\mathrm{sm}}\left(\lambda \right)\right]=k_{0}\left(\lambda \right)+\Delta k\left(\lambda \right)$$

where *k*_0_(*λ*) is its coherent light approximation computed by optimized curve fitting over its approximated values *k*_c_{*Λ*_t_} computed from *T*_sm_(*λ*) by solving Equation (1), and Δ*k*(*λ*) ≥ 0 is a partially coherent light correction [29]. However, *k*(*λ*) has been most often computed from *T*_+_(*λ*) [27,33], e.g., by solving Equation (2) using numerical integration.

The following fitting functions *Ff* were used for deriving the optimized curve fitted *n*(*λ*) and *k*_0_(*λ*) in [29]:

(6) $$\left\{ \begin{array}{l} 1).\mathrm{For}\;\mathrm{fitting}\;n\left(\lambda \right):Ff=\mathrm{Polynomial}\;\mathrm{of}\;\mathrm{optimized}\;\mathrm{degree}\;p_{o}\leq N_{f}-1\\ \mathrm{providing}\;\min\left[Fr\left(p\right)/\left(N_{f}-1-p\right)\right],\mathrm{where}:\\ N_{f}-1_{}\mathrm{is}\;\mathrm{the}\;\mathrm{number}\;\mathrm{of}\;\mathrm{tangency}\;\mathrm{wavelengths}\;\lambda_{t},\;p\mathrm{is}\;\mathrm{the}\;\mathrm{polynomial}\;\mathrm{degree},{}_{}\\ Fr{\left(p\right)}_{}\mathrm{is}\;\mathrm{the}\;\mathrm{sum}\;\mathrm{of}\;\mathrm{the}\;\mathrm{square}\;\mathrm{of}\;\mathrm{the}\;\mathrm{residuals}\;\mathrm{over}\;\mathrm{the}\;\mathrm{set}\left\{\Lambda_{f}\right\}\mathrm{for}\;\mathrm{the}\;\mathrm{polynomial}\\ \mathrm{of}\;\mathrm{degree}\;p,{}_{}e.g.Fr\left(n\left\{\Lambda_{f}\right\},p\right)=\sum_{f=1}^{N_{f}}{\left[Ff\left(n\left\{\Lambda_{f}\right\}\right)-n_{0}\left\{\Lambda_{f}\right\}\right]}^{2}{;}_{}{}_{}\\ 2).\mathrm{For}\;\mathrm{fitting}\;k_{0}\left(\lambda \right){:}_{}Ff=\mathrm{Two}-\mathrm{terms}\;\mathrm{exponential}=u_{1}\exp\left(u_{2}\lambda \right)+u_{3}\exp\left(u_{4}\lambda \right),\\ \mathrm{where}\;u_{1},u_{2},u_{3},u_{4}\mathrm{are}\;\mathrm{adjustable}\;\mathrm{parameters}. \end{array}\right.$$

The *Ff*s from Equation (6) were suitable enough and contained sufficient number of parameters, providing smooth dependencies *n*(*λ*) and *k*_0_(*λ*) for the thin films characterized in [29] by the AOEM.

After a given thin film characterization is completed, its quality can be assessed by computation of a reconstructed transmittance spectrum *T*_r_(*λ*), setting all characterization results in the right side of Equation (1), and its comparison with *T*(*λ*). A measure of the resemblance of *T*_r_(*λ*) to *T*(*λ*) is the figure of merit:

(7) $$FOM={\sqrt{\frac{\sum_{j=1}^{N_{j}}{\left\{T{\left[\lambda (j)\right]}_{}{-}_{}T_{r}\left[\lambda (j)\right]\right\}}^{2}}{N_{j}}}}_{}\geq_{}0$$

with summation over all λ⊂[min(λ_t_),λ_t_(1)], as smaller *FOM* corresponds to more accurate film characterization.

Based on using *FOM* from Equation (7), it was shown in [28] that the OEM provides more accurate film characterization compared to the FEM and the IEM, for all specimens studied there. Furthermore, *FOM*s were compared in [34] for characterizations of sputtered a-Si thin films with dissimilar average thicknesses by the OEM, the optimizing graphical method (OGM) [35], the Tauc–Lorentz–Urbach model method (TLUM) [36], and the Cody–Lorentz–Urbach model method (CLUM) [37]. These four film characterization methods were selected as most likely to provide accurate characterization of the a-Si films. The results from [34] showed that the OEM provided most accurate characterization of the films, among the four different characterization methods. The superior performance of the OEM was explained considering that it does not assume particular band tails shapes, unlike the TLUM and CLUM, and it does not assume existence of a wide spectral region of film transparency as an initial approximation, unlike the OGM [34]. Importantly, the accuracy of characterization of the same a-Si films was further increased in [29] by using the AOEM, compared to the OEM from [28].

A main problem regarding the OEM and the AOEM is that they were employed for characterization only of a-Si films on glass substrates, for *λ* ≤ 2500 nm where the substrate absorption is relatively small. In this paper, three plasma deposited films As_x_Te_100–x_ are characterized based on using the AOEM and *T* (*λ* = 300 to 3000 nm), accounting for the significant glass substrate absorption for *λ* > 2500 nm hindering accurate characterization. Description of the preparation and some composition–structure–property relationships for these glassy films were reported in [38], however data about their characteristics $\bar{d}$, ∆d, *n*(*λ*), and *k*(*λ*) have not been published, as As_x_Te_100–x_ films are insufficiently studied, in general. A simple approach is also proposed and employed for increasing the accuracy of computation of the extinction coefficient *k*(*λ*) of thin films, in comparison with its commonly used computation from *T*_+_(*λ*).

## 2. Materials and Methods

### 2.1. An Alternative Approach for Computing k (λ)

As already mentioned, *k*(*λ*) in the EMs has been computed by using equation for either *T*_sm_(*λ*) or *T*_+_(*λ*), as both equations have been derived assuming the light passing through the film to be coherent. However, it was also commented that *T*_sm_(*λ*) and especially *T*_+_(*λ*) can be influenced by partial coherence of the light due to light scattering from the film. In this regard, it was pointed out in [19,39] that the dependence:

(8) $$T_{i}(\lambda )=\sqrt{{}_{}T_{+}{\left(\lambda \right)}_{}T_{-}(\lambda )}$$

represents the interference free transmittance of the specimen. Therefore, *T*_i_(*λ*) does not depend on whether the light passing through the film is coherent or partially coherent. Moreover, an accurate formula for *T*_−_(*λ*) is derived by replacing

$\varphi_{}=4\pi n\left[\bar{d}+\left(d-\bar{d}\right)\right]/\lambda \overset{\mathrm{for}{}_{}T_{-}(\lambda )}{\underset{\mathrm{from}\mathrm{Eq}._{}(3)}{\to }}=2\pi .(\mathrm{half}-\mathrm{integer})+4\pi n\left(d-\bar{d}\right)/\lambda$ in Equation (1), as:

(9) $$T_{-}\left(\lambda \right)=\frac{1}{\varphi_{2_{-}}-\varphi_{1_{-}}}\int_{\varphi_{1_{-}}}^{\varphi_{2_{-}}}T_{u}(\varphi_{-})d\varphi_{-}{}_{}=\frac{{\left(\tau_{a,f}\tau_{f,s}\tau_{s,a}\right)}^{2}x_{s}}{\varphi_{2}{}_{{}_{-}}-\varphi_{1_{-}}}\int_{\varphi_{1_{-}}}^{\varphi_{2_{-}}}\frac{x_{}d\varphi_{-}}{a_{1}-b_{1}\cos\left(\varphi_{-}\right)+c_{1}\sin\left(\varphi_{-}\right)},$$

where $\varphi_{-}=4\pi n(d-\bar{d})/\lambda +\pi ,\varphi_{1_{-}}=-4\pi n\Delta d/\lambda +\pi ,\varphi_{2_{-}}=4\pi n\Delta d/\lambda +\pi_{}.$ Furthermore, the accuracy of computation of *T*_i_(*λ*) should be higher than that of *T*_+_(*λ*) and *T*_−_(*λ*), whose errors are usually with opposite signs due to partial coherence of the light passing through the film and *T*_i_(*λ*) is significantly smoother than *T*_sm_(*λ*).

Taking into account the above comments from this Section, replacing the known values of *T*_+_(*λ*), *T*_−_(*λ*), $\bar{d}$, ∆d, and *n*(*λ*) in Equation (8), followed by its solution, can provide the unknown *k*[*T*_i_(*λ*)] of the film for every *λ*. In film characterization based on the AOEM, since different *k*(*λ*) are computed e.g., from *T*_sm_(*λ*) and Equations (1) and (5), *T*_+_(*λ*) and Equation (2), and *T*_i_(*λ*) and Equation (8), the extinction coefficient of the film is selected to be *k*(*λ*) corresponding to the smallest *FOM* from Equation (7) [29].

### 2.2. Dual Transformation Regarding T_sm_ (λ) Taking into Account the Substrate Absorption

Although UV/VIS/NIR spectrophotometers provide light with *λ* ≈ 3000 nm [40,41], characterization of a film on glass substrate has not been performed by EM for *λ* > 2500 nm, as far as we know, since *T*(*λ* > 2500 nm) is significantly distorted due to large absorption in the glass. In order to characterize a film on a glass substrate including the region *λ* > 2500 nm, in this study is proposed and employed a dual transformation of transmittance spectra, consisting of a forward transformation and a reverse transformation.

In regard to the above, *T*_sm_(*λ*) from Equation (1), *T*_+_(*λ*) from Equation (2), *T*_−_(*λ*) from Equation (9), and *T*_i_(*λ*) from Equation (8) are proportional to the first degree of the substrate absorbance 0 < *x*_s_(*λ*) ≤ 1, as both *x*_s_(*λ*) and *T*_s_(*λ*) of glass substrate can be significantly smaller at *λ* > 2500 nm. Accordingly, the proposed forward transformation includes calculation of *T*(*λ*)’ *= T*(*λ*)/*x*_s_(*λ*) and its smoothing providing *T*_sm_(*λ*)’, which represents an approximation of the transmittance of the specimen whose substrate is replaced by a transparent one. Next, the envelopes *T*_+_(*λ*)’ and *T*_−_(*λ*)’ of *T*_sm_(*λ*), and *T*_i_(*λ*)’ = [*T*_+_(*λ*)’*T*_−_(*λ*)’]^0.5^ are computed based on the algorithm from [30], and all their tangency wavelengths *λ*_t_(i) with *T*_sm_(*λ*)’ are determined, completing the forward transformation of transmittance spectra. Thereafter, the first stage of the AOEM is executed by using the points *T*_+_(*λ*_t_)’ and *T*_−_(*λ*_t_)’. However, excluding the several longest *λ*_t_ for which *x*_s_(*λ*_t_) << 1 since *x*_s_(*λ*) is present not only in the numerator of Equations (2) and (9), and substituting the film absorbance by *x*_s_(*λ*_t_)’ = *x*_s_(*λ*_t_)/*x*_s_(*λ*_t_) = 1. At the end of such first stage of the AOEM, $\bar{d}$, ∆d ≥ 0 and *m*_1_ are computed as in [28,29].

In the reverse transformation, *T*_sm_(*λ*) *= T*_sm_(*λ*)’*x*_s_(*λ*) are calculated, its envelopes *T*_+_(*λ*) = *T*_+_(*λ*)’*x*_s_(*λ*) and *T*_−_(*λ*) = *T*_−_(*λ*)’*x*_s_(*λ*), and *T*_i_(*λ*) = *T*_i_(*λ*)’*x*_s_(*λ*). Importantly, the tangency wavelengths of *T*_sm_(*λ*) are the same as the already determined *λ*_t_(i) since *T*_sm_, *T*_+_ and *T*_−_ are represented by multiplying their respective *T*_sm_’, *T*_+_’ ≥ *T*_sm_’ and *T*_−_’ ≤ *T*_sm_’ by the same *x*_s_ > 0, for every *λ*. After *T*_sm_(*λ*), *T*_+_(*λ*), *T*_−_(*λ*), *T*_i_(*λ*) and all *λ*_t_(i) are computed, the second stage of the AOEM can be executed as in [29] taking into account the substrate absorbance *x*_s_(*λ*) ≤ 1.

### 2.3. Calculation of a Lower Limit of n(λ)

An expression about the refractive index of a uniform film on a non-transparent substrate was presented in [35]:

(10) $$n(\lambda )={\sqrt{M_{1}+\sqrt{M_{1}{}^{2}-n_{s}{}^{2}}}}_{}{}_{},{{}_{}}_{}\mathrm{where}{}_{}M_{1}(\lambda )=2n_{s}\frac{T_{u+}-T_{u-}}{T_{u+}T_{u-}}+{\frac{n_{s}{}^{2}+1}{2}}_{},$$

as *T*_u+_(*λ*) and *T*_u−_(*λ*) are the upper and lower envelopes of the smoothed transmittance spectrum of the respective specimen with uniform film.

Furthermore, the following relations are valid for a specimen with the same characteristics except that the film, with the same $\bar{d}$, is non-uniform:

(11) $$T_{+}\left(\lambda \right)\leq T_{u+}\left(\lambda \right),T_{-}\left(\lambda \right)\geq T_{u-}\left(\lambda \right)$$

where *T*_+_(*λ*) and *T*_−_(*λ*) are the upper and lower envelopes of the smoothed transmittance spectrum *T*_sm_(*λ*) of this specimen with non-uniform film [29,39]. Moreover, let us consider the quantity:

(12) $$n_{l}(\lambda )={\sqrt{M_{2}+\sqrt{M_{2}{}^{2}-n_{s}{}^{2}}}}_{}{}_{},{{}_{}}_{}\mathrm{where}{}_{}M_{2}(\lambda )=8n_{s}{}^{2}\frac{\frac{T_{+}-T_{-}}{T_{+}T_{-}}}{\frac{{\left(n_{s}+1\right)}^{2}}{x_{s}}-{\left(n_{s}-1\right)}^{2}x_{s}}+{\frac{n_{s}{}^{2}+1}{2}}_{}.$$

It is deduced from Equations (10)–(12) that:

(13) $$n{\left(\lambda \right)}_{}\geq_{}n_{l}(\lambda ),\;$$

showing that *n*_l_(*λ*) represents a lower limit of *n*(*λ*).

## 3. Results

### 3.1. Measurements Regarding the Studied As_x_Te_100–X_ Films

The chemical composition and thickness of the films have been measured by energy-dispersive X-ray microanalysis (EDS) and scanning electron microscope (SEM) using JSM IT-300LV SEM of JEOL with an energy-dispersive attachment X-MaxN 20 of Oxford Instruments at high vacuum and an accelerating voltage of 20 kV. The compositions of the three studied films have been determined as As_40_Te_60_, As_80_Te_20_, and As_98_Te_2_ in [38].

X-ray diffraction (XRD) of film on glass substrate specimens are performed on a Shimadzu XRD-7000 X-ray diffractometer at a fixed time in the range of 10–80° with a step of 0.02°, using CuKα radiation with a wavelength of 1.5406 nm and scanning speed of 2° per minute. The XRD image for the As_40_Te_60_ film on a glass substrate from Figure 1b shows amorphous structure of the film, since the image does not contain any sharp peaks inherent to crystallites. Besides, there are two halos in the XRD image from Figure 1b, the stronger in the range of 2θ = 20–40°, and the weaker at 40–60°. The presence of these features presumably indicates presence of tellurium in the form of chains, and a small amount of AsTe_3/2_ trigonal pyramidal structural units, as confirmed by Raman spectroscopy data in [38]. Furthermore, the As_80_Te_20_ and As_98_Te_2_ films also have amorphous structures revealed by similar XRD images.

**Figure 2:**
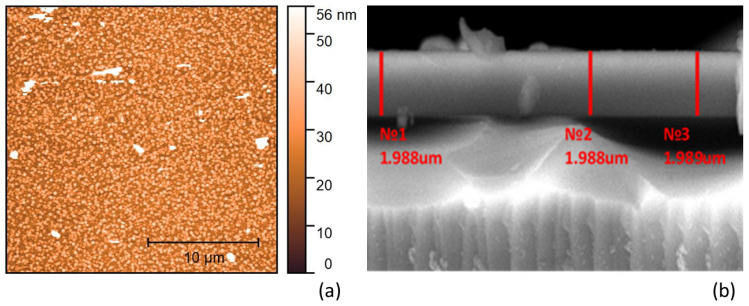
Surface and cross-section images of the As_98_Te_2_ film: (**a**) AFM picture; (**b**) scanning electron microscope (SEM) photo.

The surface morphology of the films was monitored at atmospheric conditions using Smena atomic force microscopy (AFM) head, based on the NTEGRA Spectra using HA_NC and HA_C probes NT-MDT (Zelenograd, Russia), in contact and semi-contact mode. Two-dimensional image of the surface and cross-section of the As_98_Te_2_ film are shown in Figure 2. The surface root-mean-square roughness *R*_q_ is determined from AFM image by Gwyddion open software [38]. *R*_q_ is 7.7 ± 0.7 nm for the As_40_Te_60_ film [38], 6.6 ± 0.7 nm for the As_80_Te_20_ film [38], and 8.1 ± 0.7 nm for the As_98_Te_2_ film from Figure 2a. Unfortunately, the As_40_Te_60_ and As_80_Te_20_ films have been destroyed without independent measurement of their average film thickness $\bar{d}$.

A sample containing the As_40_Te_60_ film has been prepared by five minutes deposition onto 0.45 mm thick sapphire plate with double-sided polishing manufactured by Monocrystal [38,42]. Samples including the As_80_Te_20_ film or the As_98_Te_2_ film have been formed by deposition onto 1 mm thick standard microscope slide glass substrate of Levenhuk [38,43]. Normal incidence transmittance spectra *T*(*λ*) and *T*_s_(*λ*) of these three samples and their substrates, as well as reflectance spectra *R*_s_(*λ*) of the substrates have been measured by a Cary 5000 double-beam spectrophotometer of Agilent [44]. These transmittance spectra measurements have been implemented at room temperature, with a step of 1 nm, slit width of 3.44 nm, and a circular light spot with 1 mm diameter [38].

The substrate characteristics *n*_s_(*λ*) and *k*_s_(*λ*) are computed by solving the system of two equations about *T*_s_(*λ*) and *R*_s_(*λ*), for every *λ*, as in [27]. Every smoothed spectrum *T*_sm_(*λ*) of a studied sample is computed by “external smoothing” of its *T*(*λ*), as described in the fifth paragraph of the Introduction and in [29]. Data and results regarding the characterization of the three As_x_Te_100–x_ films based on the AOEM are presented in the next three figures, whereas the results referring to *λ*_t_(i) are exhibited by open circles, and the most important computed results are in red color. The optimized curve fittings for deriving *n*(*λ*) and *k*_0_(*λ*) are performed as described in Equation (6).

### 3.2. Characterization of the As_40_Te_60_ Film on Sapphire Substrate by the AOEM

**Figure 3:**
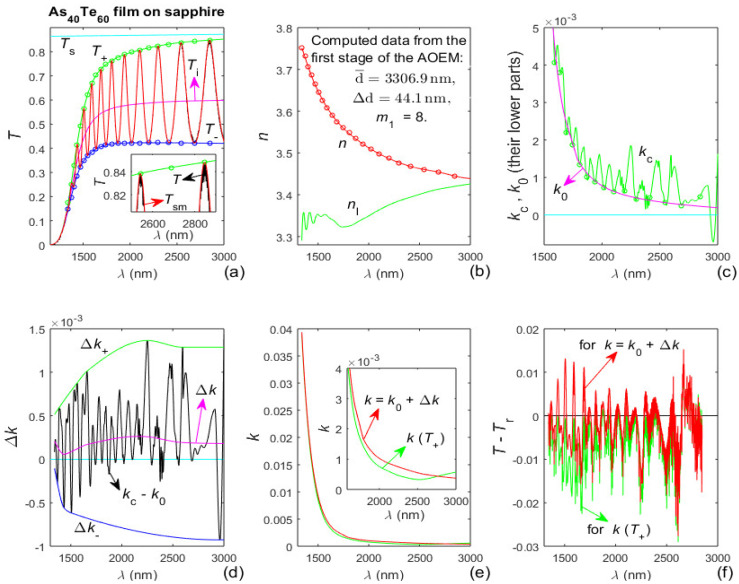
Input data and computed results from the characterization of the As_40_Te_60_ film by the advanced optimizing envelope method (AOEM). (**a**) *T*_s_(*λ*), *T*(*λ*), *T*_sm_(*λ*), its envelopes *T*_+_(*λ*) and *T*_−_(*λ*), the points *T*_+_(*λ*_t_) and *T*_−_(*λ*_t_) represented by circles, and *T*_i_(*λ*); (**b**) *n*(*λ*) determined by optimized curve fitting over *n*_0_(*λ*_t_) visualized by circles, and its lower limit *n*_l_(*λ*); (**c**) *k*_c_(*λ*) obtained from Equation (1) and its optimized curve fitted *k*_0_(*λ*); (**d**) deriving the partially coherent light correction Δ*k*(*λ*) > 0; (**e**) *k*(*T*_+_) computed from Equation (2) and *k* = *k*_0_ + Δ*k*; (**f**) difference between *T*(*λ*) and *T*_r_(*λ*) computed using *k*(*T*_+_) and *k* = *k*_0_ + Δ*k*, respectively.

Since sapphire is quasi-transparent over the used interval of *λ* [45], its *x*_s_(*λ*) ≅ 1, *T*_s_(*λ*)~*x*_s_(*λ*) ≅ constant [27], and the AOEM is executed as in [29], except for absence of adjustment of the envelopes *T*_+_(*λ*) and *T*_−_(*λ*) for substrate absorption. Data and characterization results for the As_40_Te_60_ film are shown in Figure 3. The values of $\bar{d}$, ∆d ≥ 0 and *m*_1_ computed at the end of the first stage of the AOEM are typed in Figure 3b.

It is seen from Figure 3b that the refractive index *n*(*λ*) of the film decreases with increasing *λ*, i.e., it has a normal dispersion in the whole studied spectral range, and *n*(*λ*) is larger than its lower limit *n*_l_(*λ*) in accordance with Equation (13). Furthermore, Figure 3c,e show that the film has a wide spectral region of quasi-transparency, i.e., a region with negligibly small *k*(*λ*).

### 3.3. Characterization of the As_98_Te_2_ Film on Glass Substrate by the AOEM

**Figure 4:**
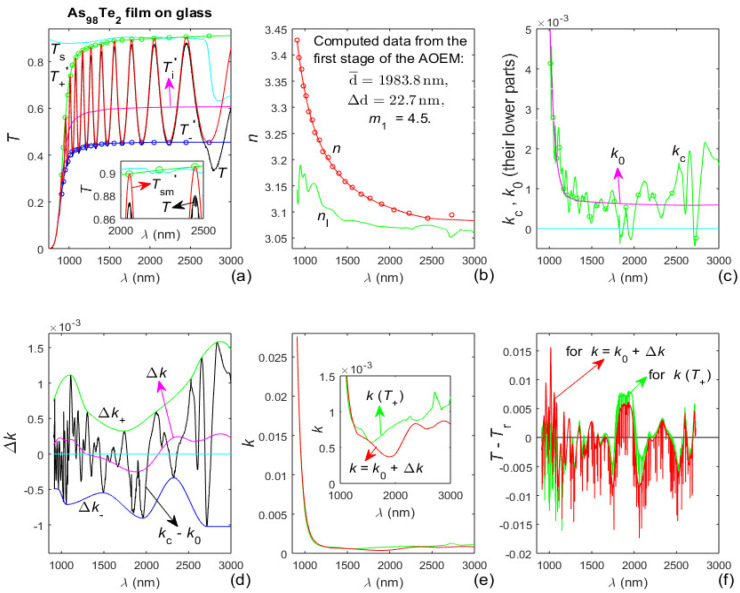
Input data and computed results from the characterization of the As_98_Te_2_ film by the AOEM. (**a**) *T*_s_(*λ*), *T*(*λ*), *T*_sm_(*λ*)’, its envelopes *T*_+_(*λ*)’ and *T*_−_(*λ*)’, the points *T*_+_(*λ*_t_)’ and *T*_−_(*λ*_t_)’ represented by circles, and *T*_i_(*λ*)’; (**b**) *n*_0_(*λ*_t_) shown by circles, *n*(*λ*), and its lower limit *n*_l_(*λ*); (**c**) *k*_c_(*λ*) and its optimized curve fitted *k*_0_(*λ*); (**d**) deriving the partially coherent light correction Δ*k*(*λ*); (**e**) *k*(*T*_+_) computed from Equation (2) and *k* = *k*_0_ + Δ*k*; and (**f**) difference between *T*(*λ*) and *T*_r_(*λ*) computed using *k*(*T*_+_) and *k* = *k*_0_ + Δ*k*, respectively.

The As_98_Te_2_ film has been deposited for fifteen minutes on a glass substrate described in Section 3.1 [38]. Such glass substrates are strongly absorbing for *λ* > 2500 nm as revealed by the significantly lower values of *T*_s_(*λ* > 2500 nm) from Figure 3a. Therefore, the dual transformation regarding *T*_sm_(*λ*), described in Section 2.2 is employed, as the first stage of the AOEM is implemented excluding only the longest *λ*_t_(i) since only it is within the region of significantly lower values of *T*_s_(*λ*). Data and results from the characterization of the As_98_Te_2_ film by the AOEM are exhibited in Figure 4.

It is seen from Figure 4a that *T*_sm_(*λ*)’, *T*_+_(*λ*)’ and *T*_−_(*λ*)’, obtained by the forward transformation described in Section 2.2, do not change their appearance in the region *λ* > 2500 nm of strong absorption in the substrate, unlike the significantly lower *T*_s_(*λ*) and *T*(*λ*) there. This allows accurate computation of *T*_sm_(*λ*)’, *T*_+_(*λ*)’, *T*_−_(*λ*)’, *T*_i_(*λ*)’ and *λ*_t_(i); followed by accurate computation of *T*_sm_(*λ*), *T*_+_(*λ*), *T*_−_(*λ*) and *T*_i_(*λ*) by the reverse transformation described in Section 2.2. Figure 4b shows that *n*(*λ*) has a normal dispersion in the whole studied spectral range and *n*(*λ*) > *n*_l_(*λ*). Besides, the film also has a wide spectral region of quasi-transparency-Figure 4c,e. Notably, the difference between the average film thickness $\bar{d}$ = 1983.8 nm from Figure 4b computed by the AOEM and $\bar{d}$ = 1988.3 nm from the SEM image from Figure 2b is 0.24%.

The positive partially coherent light correction Δ*k*(*λ*) ≈ 2 × 10^−4^ for the As_40_Te_60_ film, seen from Figure 3d, is attributed to light scattering from this relatively thick film resulting in slight shrinkage of the interference pattern of *T*(*λ*) [29]. The positive or negative Δ*k*(*λ*) for the As_98_Te_2_ film from Figure 4d is ascribed to smaller light scattering from this thinner film being sufficiently counterbalanced by the external smoothing of *T*(*λ*).

### 3.4. Characterization of the As_80_Te_20_ Film on Glass Substrate Based on the AOEM

**Figure 5:**
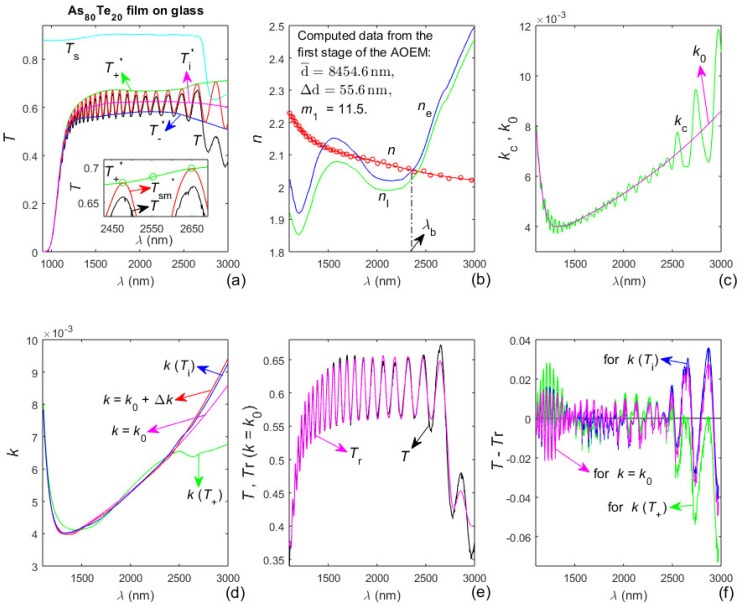
Input data and computed results from the characterization of the As_80_Te_20_ film by the AOEM. (**a**) *T*_s_(*λ*), *T*(*λ*), *T*_sm_(*λ*)’, its envelopes *T*_+_(*λ*)’ and *T*_−_(*λ*)’, and *T*_i_(*λ*)’; (**b**) *n*_0_(*λ*_t_), *n*(*λ*), *n*_l_(*λ*) and *n*_e_(*λ*); (**c**) *k*_c_(*λ*) and its optimized curve fitted *k*_0_(*λ*); (**d**) *k*(*T*_+_) computed from Equation (2), *k*(*T*_i_) from Equation (8) and *k* = *k*_0_ + Δ*k*; (**e**) *T*(*λ*) and the reconstructed spectrum *T*_r_(*λ*) computed using *k* = *k*_0_; and (**f**) difference between *T*(*λ*) and *T*_r_(*λ*) with *k* = *k*_0_, *k*(*T*_+_) and *k*(*T*_i_).

The As_80_Te_20_ film has been deposited for thirty minutes on a glass substrate described in Section 3.1 [38]. The dual transformation regarding *T*_sm_(*λ*) is implemented, as the first stage of the AOEM is executed excluding the four longest *λ*_t_(i) since they are within the region of significantly lower values of *T*_s_(*λ*) from Figure 5a. Data and results from the characterization of the As_80_Te_20_ film by the AOEM are presented in Figure 5, as the optimized number of *λ*_t_(i) participating in the first stage of the characterization is *n* = 26. The dependence *n*_e_(*λ*) from Figure 5b is determined by substituting the already computed *T*_+_(*λ*), *T*_−_(*λ*), $\bar{d}$, ∆d, and *m*_1_ in Equations (2) and (9) of the envelopes. Solving these two equations with respect to the two unknown *n*_e_ and *k*_e_ components of the complex refractive index of the film, for every *λ*. The longest wavelength of *n*(*λ*) crossing *n*_e_(*λ*) is denoted by *λ*_b_ in Figure 5b.

A comparison of *T*(*λ*) from Figure 3a, Figure 4a and Figure 5a shows that the As_80_Te_20_ film has quite different optical characteristics with respect to the As_40_Te_60_ and As_98_Te_2_ films. For example, the much narrower interference pattern of *T*(*λ*) from Figure 5a demonstrates that the refractive index *n*(*λ*) of the As_80_Te_20_ film is significantly smaller than that of the As_40_Te_60_ and As_98_Te_2_ films, as explained in [19]. Also, the more than twice larger number N_e_ of extrema of *T*_sm_(*λ*) from Figure 5a indicates that the product *n*(*λ*)$\bar{d}$ for the As_80_Te_20_ film is at least twice larger than that for the As_40_Te_60_ and As_98_Te_2_ films, as implied by Equation (3). The above data show that the average thickness of the As_80_Te_20_ film is significantly larger than 2$\bar{d}$ = 6613.8 nm of the As_40_Te_60_ film.

Also, the difference *T*_s_(*λ*) − *T*_+_(*λ*) is much larger in the region of the interference pattern from Figure 5a than for the As_40_Te_60_ and As_98_Te_2_ films, indicating significantly stronger absorption and larger *k*(*λ*) of the As_80_Te_20_ film. Moreover, *T*_i_(*λ*) from Figure 5a decreases with increasing *λ* above 2300 nm implying increasing absorption there, which can be attributed to anomalous dispersion in this region. Besides, the difference *T*_+_(*λ*) − *T*_−_(*λ*) from Figure 3a and Figure 4a does not have an apparent minimum in the region of the interference pattern. However, *T*_+_(*λ*) − *T*_−_(*λ*) regarding Figure 5a rises with increasing *λ* above its apparent minimum at *λ* ≈ 2100 nm, indicating increasing *n*(*λ*) for *λ* > 2100 nm, thus confirming the presence of anomalous dispersion within the studied spectral region of the As_80_Te_20_ film.

Furthermore, it is seen from Figure 5b that the respective characterization of the As_80_Te_20_ film by the AOEM does not recognize the commented above presence of anomalous dispersion. It is also apparent from Figure 5b that the difference *n*_l_(max(*λ*)) − *n*(max(*λ*)) is too large, as *n*(*λ* > *λ*_b_) < *n*_e_(*λ* > *λ*_b_) ≈ *n*_l_(*λ* > *λ*_b_) although *n*_l_(*λ*) should be a lower limit of *n*(*λ*) according to Equation (13). Moreover, the difference |*T*(*λ* > 2600 nm) − *T*_r_(*λ* > 2600 nm)| reaches too large values in Figure 5f compared to Figure 3f and Figure 4f. These facts show that the represented in Figure 5 characterization of the As_80_Te_20_ film by the AOEM is inaccurate in the region of anomalous dispersion of the film. A main reason for this is the exclusion of the four longest *λ*_t_(i) from the execution of the first stage of the AOEM characterization corresponding to Figure 5, taking into account that these *λ*_t_(i) also belong to the region of anomalous dispersion where *T*_+_(*λ*) − *T*_−_(*λ*) rises significantly with increasing *λ*.

Importantly, accurate film characterization by any EM requires determination of the correct first interference order *m*_1_, which has integer or half-integer value, since it imposes a narrow interval of possible values of the average film thickness $\bar{d}$ in accordance with Equation (3). Correspondingly, the failure of the characterization of the As_80_Te_20_ film by the AOEM represented by Figure 5 to recognize the presence of anomalous dispersion is attributed to determination of incorrect *m*_1_.

Aiming at selection of the correct *m*_1_, a simplified AOEM is employed by fixing both *m*_1_ to every possible value from Equation (3) and the number of used extrema of *T*_sm_(*λ*) to N_e_, for each one of the three films. At the second stage of the respective simplified characterizations of the film, *k*(*T*_i_) computed from Equation (8) is used as described in Section 2.1. The main results from these computations are given in Table 1.

It is seen from Table 1 that the smallest δd/*n* and *FOM* correspond to the same *m*_1_ for each one of the As_40_Te_60_ and As_98_Te_2_ films, thus confirming the accuracy of their characterizations represented by Figure 3 and Figure 4. Furthermore, this justifies the main concept of the OEM and the AOEM that computation of $\bar{d}$, ∆d, and *m*_1_ corresponding to the smallest δd/*n* is required for accurate computation of *n*(*λ*) and *k*(*λ*) of the characterized thin dielectric or semiconductor film with $\bar{d}$ = (300,5000) nm.

**Figure 6:**
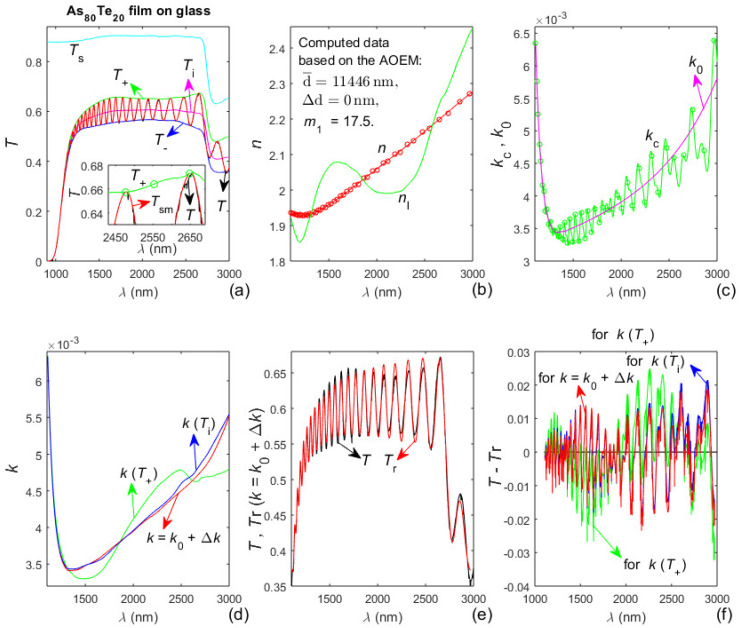
Input data and computed results from the characterization of the As_80_Te_20_ film based on the AOEM with $\bar{d}$, ∆d, and *m*_1_ listed in red in Table 1. (**a**) *T*_s_(*λ*), *T*(*λ*), *T*_sm_(*λ*), its envelopes *T*_+_(*λ*) and *T*_−_(*λ*), and *T*_i_(*λ*); (**b**) *n*(*λ*) and *n*_l_(*λ*); (**c**) *k*_c_(*λ*) and its optimized curve fitted *k*_0_(*λ*); (**d**) *k*(*T*_+_) computed from Equation (2), *k*(*T*_i_) from Equation (8) and *k* = *k*_0_ + Δ*k*; (**e**) *T*(*λ*) and *T*_r_(*λ*) computed using *k* = *k*_0_ + Δ*k*; and (**f**) difference between *T*(*λ*) and *T*_r_(*λ*) with *k*(*T*_i_), *k*(*T*_+_) and *k* = *k*_0_ + Δ*k*.

However, the smallest δd/*N* and the smallest *FOM* correspond to different values of *m*_1_ for the significantly thicker As_80_Te_20_ film. Since the film characterization should provide reconstructed transmittance spectrum *T*_r_(*λ*) as close as possible to *T*(*λ*), another characterization of the As_80_Te_20_ film is performed based on using the AOEM with the values of $\bar{d}$, ∆d, and *m*_1_ listed in red in Table 1. Data and results from this characterization are shown in Figure 6.

Notably, it is seen from Figure 6b that its corresponding characterization recognizes the presence of anomalous dispersion for the As_80_Te_20_ film, as the difference *n*_l_(max(*λ*)) − *n*(max(*λ*)) is significantly smaller than for the characterization from Figure 5. This warrants the procedure for AOEM characterization of films thicker than 5000 nm, by selection of *m*_1_ based on determination of min{*FOM* (*m*_1_,*k*(*T*_i_))}, exemplified by the use of Table 1 for the As_80_Te_20_ film.

### 3.5. Additional Results about the As_x_Te_100−X_ Films

Other data regarding characterizations of the three studied films based on the AOEM are presented in Table 2, as the data about the As_40_Te_60_ and As_98_Te_2_ films refer to the characterizations represented by Figure 3 and Figure 4. The data related to the As_80_Te_20_ film and designated as “*m*_1_ = 11.5, ND” and “*m*_1_ = 17.5, AD” refer to the characterizations represented by Figure 5 and Figure 6 respectively, as “ND” denotes normal dispersion and “AD”—anomalous dispersion. Since *n*(*λ*) from Figure 5b does not demonstrate anomalous dispersion and its respective *n*_l_(max(*λ*)) − *n*(max(*λ*)) is quite large, one more characterization of the As_80_Te_20_ film is performed using the film data typed in Figure 5b and *n*(*λ* ≤ *λ*_b_) from Figure 5b, however employing *n*(*λ* > *λ*_b_) = *n*_e_(*λ* > *λ*_b_). Taking into account that this characterization recognizes the presence of anomalous dispersion, it is denoted as “*m*_1_ = 11.5, AD”. However, the *FOM* data from Table 2 show that this particular characterization is most inaccurate amongst the three represented there characterizations of the As_80_Te_20_ film.

Importantly, a comparison of the data from the fourth and fifth columns of Table 2 shows that the extinction coefficient of the film is computed more accurately from *T*_i_(*λ*) and Equation (8) rather than from *T*_+_(*λ*) and Equation (2), for all five film characterizations based on the AOEM. This is mainly attributed to the independence of *T*_i_(*λ*) from possible partial coherence of the light passing through the film and light scattering from the film, as discussed in Section 2.1.

**Figure 7:**
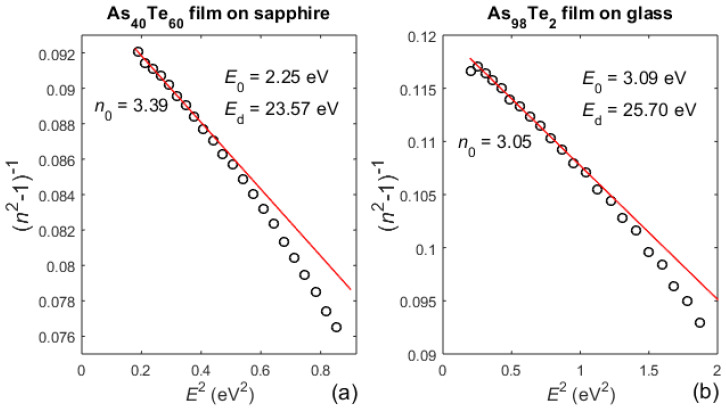
Wemple–DiDomenico (WD) plots. (**a**) for the As_40_Te_60_ film, (**b**) for the As_98_Te_2_ film. The low-energy linear regression to the WD plot is represented by red line. *n*{min(*E*(*λ*_t_))} is disregarded from the linear regression from Figure 7b due to its arguable inaccuracy, since only max(*λ*_t_) is in the region of strong substrate absorption.

The Wemple–DiDomenico (WD) single-effective-oscillator approximation $n(E)≃\sqrt{1+\frac{E_{0}E_{d}}{E_{0}{}^{2}-E^{2}}}$ is known to be valid for amorphous semiconductors and glasses, where *E*_0_ is the oscillator energy, *E*_d_ is the dispersion energy, and *E*(eV) = 1.2398/*λ* (µm) is the photon energy [46]. Regardingly, in Figure 7 are presented WD plots {*n*(*E*(*λ*_t_))^2^−1}^−1^ versus *E*(*λ*_t_)^2^ [46] for the As_40_Te_60_ and As_98_Te_2_ films. The parameters *E*_0_ and *E*_d_ are determined by a low-energy linear regression to the WD plot, and the static refractive index is $n_{0}=n(E=0)\;≃\;\sqrt{1+E_{d}/E_{0}}$.

The dispersion energy, Ed, measures the average strength of the interband optical transitions, and has been found to obey the empirical relationship [46]:

(14) $$E_{d}\left(\mathrm{eV}\right)=\beta N_{c}Z_{a}N_{v}$$

where *β* = *β*_c_ = 0.37 ± 0.04 eV in ‘covalent’ materials, *β* = *β*_io_ = 0.26 ± 0.03 eV in ‘ionic’ materials, *N*_c_ is the coordination number of the cation nearest neighbor to the anion, *Z*_a_ is the formal chemical valence of the anion, and *N*_v_ is the total number of valence electrons (cores excluded) per anion. Using *E*_d_ typed in Figure 7, Equation (14), and *β* = *β*_c_ is estimated the coordination number of the cation (As) to be *N*_c_ ≈ 3.5 for the As_40_Te_60_ film, and *N*_c_ ≈ 3.56 for the As_98_Te_2_ film.

Furthermore, *E*_0_ for the As_80_Te_20_ film is significantly smaller than for the As_40_Te_60_ and As_98_Te_2_ films, taking into account that its *n*(*E*) is also significantly smaller in accordance with Figure 3b, Figure 4b, and Figure 6b and the WD approximation. Since min(*E*) is too close to *E*_0_ for the As_80_Te_20_ film, anomalous dispersion occurs and low-energy linear regression to the WD plot cannot be used for accurate determination of *E*_0_, *E*_d_, and *n*_0_.

## 4. Discussion

The OEM [28] was developed for increasing the accuracy of characterization of a thin film on a substrate from interference *T*(*λ*) of the specimen, by optimization of the three parameters of the IEM from [27], accounting for possible non-uniformity of the film and substrate absorption. It was demonstrated in [34] that the OEM from [28] provides most accurate characterization of a-Si films with dissimilar thicknesses compared to the OGM [35], TLUM [36], and CLUM [37] selected as the most likely methods for accurate characterization of these films. Further increasing the characterization accuracy was achieved by the AOEM [29] based on: external smoothing of *T*(*λ*) offsetting the influence of light scattering, enhanced computation of the envelopes using both iteration and extra points, and computation of *n*(*λ*) and *k*(*λ*) by optimized curve fitting.

In the first stage of the AOEM, the average thickness $\bar{d}$, the thickness non-uniformity ∆d over the light spot, and the first interference order *m*_1_ of the film are computed, by minimization of the error metric, usually δd/*N* > 0. This error metric can be used for estimation of the relative error δd/$\bar{d}$ = δd/*N.* (*N*/$\bar{d}$) in the computation of $\bar{d}$ of the characterized film. In order to compare the accuracy of computation of $\bar{d}$, in Table 3 are presented data about the relative error δd/$\bar{d}$ for the presented here characterizations of As_x_Te_100−x_ films and for recent characterizations of a-Si and As_33_S_67_ based on the FEM and the AOEM.

Notably, the films characterized based on the FEM have been developed to be uniform, deposited on quasi-transparent substrates, and $\bar{d}$ of the films from [47] has been chosen to provide neither too high nor too low values of *m*_1_, as all of these factors are favorable for accurate film characterization. Nevertheless, it is seen from Table 3 that the characterization of films based on the AOEM provides significantly smaller relative error δd/$\bar{d}$, and therefore more accurate $\bar{d}$, in comparison with the characterizations based on the FEM; the only exception being the As_80_Te_20_ film.

The record low value of δd/$\bar{d}$ = 0.101% in Table 3 is for the As_40_Te_60_ film and it is associated with the quasi-transparency of its sapphire substrate, unlike the absorption of the glass substrates. The fact that the second lowest δd/$\bar{d}$ = 0.133% from Table 3 is for the As_98_Te_2_ film is believed to be mostly due to a superior accuracy of its respective envelopes *T*_+_(*λ*) and *T*_−_(*λ*) as a result of the implementation of the proposed here dual transformation regarding *T*_sm_(*λ*).

Furthermore, the relative error in the computation of the refractive index of the film is δ*n*/*n* = δd/$\bar{d}$ + δ*λ*_t_/*λ*_t_, according to Equation (3), where δ*λ*_t_/*λ*_t_ is the relative error in computation of *λ*_t_. Justifiably assuming δ*λ*_t_/*λ*_t_ ≤ 0.15% [28,29,30] leads to δ*n*/*n* < 0.3% for the characterizations of the As_40_Te_60_ and As_98_Te_2_ films by the AOEM represented by Figure 3 and Figure 4. The significantly smaller *n*(*λ*) and larger *k*(*λ*) of the As_80_Te_20_ film are attributed to some porosity, oxidation in the film, and its non-stoichiometry in comparison with the As_40_Te_60_ and As_98_Te_2_ films. In this regard, anomalous dispersion has already been observed for other chalcogenide thin films [49].

## 5. Conclusions

The AOEM is employed for optical characterization of three As_x_Te_100−x_ films with different compositions and dissimilar $\bar{d}$, only from a normal incidence interference transmittance spectrum *T*(*λ* ≤ 3000 nm) of a specimen consisting of the film on a substrate. To cope with the strong light absorption in commonly used glass substrates for *λ* > 2500 nm, a dual transformation of *T*(*λ*) is proposed and implemented for accurate computation of its envelopes *T*_+_(*λ*) and *T*_−_(*λ*). This dual transformation is simpler and should provide more accurate envelopes of *T*(*λ*) in comparison with the computation of the envelopes from [29], in case of non-transparency of the substrate. The accuracy of computation of $\bar{d}$ is record low <0.15%, implying <0.3% accuracy of computation of *n*(*λ*) of the As_40_Te_60_ and As_98_Te_2_ films by the AOEM.

It is justified that the As_80_Te_20_ film exhibits a region of anomalous dispersion and is significantly thicker than 6600 nm, which makes it very difficult for characterization by envelope method. In this respect, a procedure is proposed for accurate determination of the first interference order *m*_1_ for such a thick film, and this procedure is used for characterization of the As_80_Te_20_ film based on the AOEM. As far as we are aware of, the As_80_Te_20_ film is the only one with anomalous dispersion and the thickest, with estimated $\bar{d}$ = 11446 nm, ever characterized by an envelope method.

It is also pointed out that *T*_i_(*λ*) does not depend on the scattering of light from the film, unlike *T*_+_(*λ*). Consequently, it is shown that computation of *k*(*λ*) from *T*_i_(*λ*) and Equation (8) is more accurate than the commonly employed computation of *k*(*λ*) from *T*_+_(*λ*) and Equation (2), for all five characterizations of As_x_Te_100−x_ films based on the AOEM and represented in Table 2.

The obtained results strengthen our conviction that the AOEM has a capacity for providing most accurate optical characterization of almost every dielectric or semiconductor film with $\bar{d}$ > 300 nm on a substrate compared to all the other methods for characterization of such films only from *T*(*λ*).

## Figures and Tables

**Table 1:** The main results from the characterizations of the three As_x_Te_100–x_ films by the simplified AOEM with fixed values of *m*_1_ and *N* = N_e_. The data corresponding to the smallest *FOM* (*k*(*T*_i_)) are in red color and the data regarding the smallest δd/*N* are in blue if they refer to different *m*_1_.

**As_40_Te_60_ Film, *m*_1_ Is Fixed, *N* = N_e_ = 22**				
**δd/*N* (nm)**	***m*_1_**	**∆d (nm)**	$\bar{d}$ **(nm)**	***FOM* (*k*(*T*_i_))**
3.63 × 10^−2^	6	47	2676.5	0.0178
1.89 × 10^−2^	7	44	2994.7	0.00982
0.85 × 10^−2^	8	38	3349.2	0.00500
1.20 × 10^−2^	9	25	3748.3	0.00560
2.36 × 10^−2^	10	1	4114.8	0.01048
**As_98_Te_2_ Film, *m*_1_ Is Fixed, *N* = N_e_ = 22**				
**δd/*N* (nm)**	***m*_1_**	**∆d (nm)**	$\bar{d}$ **(nm)**	***FOM* (*k*(*T*_i_))**
4.50	2.5	40	1355.7	0.0461
2.13	3.5	32	1671.3	0.0210
0.520	4.5	23	1981.1	0.00512
2.59	5.5	0	2286.9	0.0188
6.29	6.5	0	2523.6	0.0447
**As_80_Te_20_ Film, *m*_1_ Is Fixed, *N* = N_e_ = 46**				
**δd/*N* (nm)**	***m*_1_**	**∆d (nm)**	$\bar{d}$ **(nm)**	***FOM* (*k*(*T*_i_))**
19.83	4.5	95	4945.4	0.02441
18.03	5.5	95	5425.2	0.02265
16.43	6.5	95	5808.8	0.01909
15.11	7.5	95	5947.2	0.01544
13.83	8.5	94	6496.4	0.01398
12.79	9.5	93	6914.5	0.01243
12.10	10.5	92	7325.7	0.01130
11.80	11.5	92	7847.5	0.01035
11.92	12.5	90	8302.6	0.00981
12.27	13.5	79	9064.3	0.00937
12.14	14.5	74	9586.0	0.00898
12.11	15.5	63	10,195	0.00892
12.07	16.5	45	10,916	0.00878
11.94	17.5	0	11,446	0.00859
12.10	18.5	0	11,867	0.00889
12.63	19.5	0	12,288	0.00931

**Table 2:** Supplementary data about the characterizations of the As_x_Te_100−x_ films based on the AOEM, as *p*_0_ is the optimized degree of the polynomial representing *n*(*λ*) according to Equation (6). The lowest *FOM* for a given film is in red color and the lowest *FOM* for each of the other characterizations of the As_80_Te_20_ film is in blue. The selected extinction coefficient *k*(*λ*) of each of the three films corresponds to the *FOM* value in red color.

Film	δd/*N* (nm)	*p*_0_	*FOM* (*k*(*T*_+_))	*FOM* (*k*(*T*_i_))	*FOM* (*k*_0_(*λ*))	*FOM* (*k*_0_(*λ*) + Δ*k*(*λ*))
As_40_Te_60_	0.335	15	8.23 × 10^−3^	7.29 × 10^−3^	7.25 × 10^−3^	5.94 × 10^−3^
As_98_Te_20_	0.220	9	3.96 × 10^−3^	3.74 × 10^−3^	4.36 × 10^−3^	4.26 × 10^−3^
As_80_Te_20_, *m*_1_ = 11.5, ND	6.89	6	1.57 × 10^−2^	1.04 × 10^−2^	1.16 × 10^−2^	1.23 × 10^−2^
As_80_Te_20_, *m*_1_ = 11.5, AD	6.89	6	1.96 × 10^−2^	1.92 × 10^−2^	3.39 × 10^−2^	1.90 × 10^−2^
As_80_Te_20_, *m*_1_ = 17.5, AD	11.94	7	1.04 × 10^−2^	8.59 × 10^−3^	9.42 × 10^−3^	8.23 × 10^−3^

**Table 3:** Data regarding the relative error δd/$\bar{d}$ for the characterizations of As_x_Te_100−x_ films and for recent film characterizations based on the FEM and the AOEM. The data about characterizations based on the FEM are in black, the data for characterizations using the OEM are in blue, and the data for characterizations based on the AOEM are in red.

Film, Specimen Number	From	m_1_	N	δd/*N* (nm)	$\bar{d\;}(\mathrm{nm})$	$\delta d/\bar{d}\;(\%)$
a-Si, 029	[47]	4	12	1.010	1172.0	1.034
a-Si, 074	[47]	4	14	1.349	1269.0	1.489
a-Si, 038	[34]	2	9	0.341	785.7	0.391
a-Si, 038	[29]	2	9	0.318	774.6	0.369
a-Si, 041	[34]	12	17	0.594	3939.1	0.256
a-Si, 041	[29]	12	17	0.567	3929.9	0.245
As_33_S_67_	[48]	2	9	0.706	744.8	0.853
As_40_Te_60_	here	8	11	0.335	3306.9	0.101
As_98_Te_2_	here	4.5	12	0.220	1983.8	0.133
As_80_Te_20_	here	17.5	46	11.94	11446	4.799
